# Absolute and Relative Judgment Accuracy: Early Childhood Teachers' Competence to Evaluate Children's Mathematical Skills

**DOI:** 10.3389/fpsyg.2021.701730

**Published:** 2021-10-18

**Authors:** Georg Hosoya, Sigrid Blömeke, Katja Eilerts, Lars Jenßen, Michael Eid

**Affiliations:** ^1^Fachbereich Erziehungswissenschaft und Psychologie, Freie Universität Berlin, Berlin, Germany; ^2^Centre for Educational Measurement, University of Oslo, Oslo, Norway; ^3^Institut für Erziehungswissenschaften, Mathematik Primarstufe, Humboldt Universität zu Berlin, Berlin, Germany

**Keywords:** judgment accuracy, relative accuracy, absolute accuracy, early childhood teachers, mathematical skills, early childhood education

## Abstract

This study examined absolute and relative judgment accuracies of German early childhood (EC) teachers with respect to the mathematical skills of the children under their supervision. The two types of judgment accuracies are crucial prerequisites for pacing activities in EC education and offering differentiated educational activities adapted to individual skill levels of children. Data from 39 EC teachers and 268 children were analyzed using multilevel modeling. Teachers rated the skills of children on a structured observation instrument (“Kinder Diagnose Tool,” KiDiT). Children were assessed on their mathematical skills with a standardized test (“Mathematische Basiskompetenzen im Kindesalter,” MBK-0). On average, 65% of the variation in judgments of teachers on the KiDiT could be explained by MBK-0 scores of children, which suggest that teachers are—on average—able to rank children within their groups. Teachers were also able to judge the mathematical level of skills of children as assessed by the MBK-0. Neither mathematical content knowledge (MCK) of teachers nor their mathematics pedagogical content knowledge (MPCK) or general pedagogical knowledge (GPK) moderated the relationship between judgments of teachers and test scores of children or the relationship between the level of the judgments and the level of test scores. Conclusions for future research and practice are drawn.

## Absolute and Relative Judgment Accuracies

It is highly important for adaptive support of learning and development that teachers' abilities to judge the knowledge and skills of students are accurate (Hoge and Coladarci, 1989; Südkamp et al., 2012). Studies of primary school teachers' judgment accuracy in the domain of mathematics revealed that the accuracy of teachers was significantly positively associated with gains in mathematics achievement of students (Thiede et al., 2015). If teachers are not able to judge accurately, it is difficult for them to provide educational activities that support the learning and development of students (Thiede et al., 2018).

In early childhood (EC) education, children are younger than in primary school, and judging their knowledge and skills is particularly challenging since educational situations are multi-dimensional and less predictable than in a school context (Wickstrom et al., 2019; Pyle et al., 2020). Studies by Bruns (2014), Wullschleger (2017), Meier-Wyder (2020), and Vogler (2020) revealed specifically for German-speaking countries how much EC teachers struggle with, firstly, evaluating appropriately mathematical skills of children and then, secondly, adapting their educational activities to these. At the same time, the judgment accuracy of EC teachers has similar consequences for the mathematical development of children, for example, with respect to assignment to special education or decisions about school readiness (Gasteiger and Benz, 2018; Stillerova et al., 2019). Moreover, consequences could be long-term because early skills have to some extent predictive power for later school achievement (Duncan et al., 2007; Krajewski and Schneider, 2009; Bailey et al., 2018).

### Relevance of the Study

Studies on the judgment accuracy of EC teachers are very rare. The purpose of the present study is to close part of this research gap with respect to the mathematical skills of children in play-based EC education. In Germany, the context of the present study, play-based EC education takes place roughly up to age 5 or 6 depending on the federal state. EC teachers are responsible for small heterogeneous groups of about eight children aged 3–5 or 6 and are supposed to use naturally unfolding opportunities to support the development of knowledge and skills of children toward the aims of EC education as described in national or local guidelines. Opportunities can either unfold through free play where children select the activities or through structured play where the activity is initiated by the EC teacher (Anders and Rossbach, 2015). In many EC institutions, the following final year of EC education is regarded as a transition period to primary school where play-based activities are increasingly combined with organized instruction (hereafter referred to as “preschool”). This transition period is not included in the present study. German primary school starts after the transition period at age 6 or 7 with Grade 1.

To our knowledge, this is one of the first studies taking place in a purely play-based EC environment, before the slightly more structured transition period starts, although such studies have been encouraged for a long time (Elliott et al., 2007). The few other studies available on judgment accuracy of EC teachers took place in the context of preschool (Kilday et al., 2012; Dollinger, 2013; Kowalski et al., 2018). This means that the learning environment in these studies was still play based but increasingly pre-planned by the teacher and with stronger focus on preparing children for primary school. It is therefore questionable that this state-of-research can be transferred to purely play-based EC education.

Moreover, we were interested to learn how the judgment accuracy of teachers is related to the content knowledge, pedagogical content knowledge, and general pedagogical knowledge (GPK) of teachers. Our study goes also in this respect substantially beyond the state of research, because it used standardized measures of knowledge of EC teachers while existing studies typically used proxies, such as formal degrees or course credits (Lin and Magnuson, 2018).

## State of Research

### Judgment Accuracy: A Conceptual Framework

Cronbach's (1955) seminal article stated that judgment accuracy is not a one-dimensional construct. Commonly, two components are distinguished: relative accuracy and absolute accuracy. *Relative* accuracy can be defined “as the correspondence between the relative standing of two sets of values: (a) the judgments of teachers about their students and (b) the actual performance of students on a relevant standardized test” (Hoge and Coladarci, 1989, p. 302). The correspondence can be expressed, for instance, through correlation or regression coefficients. This type of judgment accuracy of teachers is the focus of most studies available (Südkamp et al., 2012), and it is one of the research objectives of the present paper. Since EC education represents a multi-level context where children are nested in groups, this rank component of judgment accuracy is operationalized as random slopes (see for a similar approach for pre-service primary and secondary teachers: Bonefeld et al., 2020).

In contrast, *absolute* accuracy can be defined as the difference between the level of the judgment of a teacher and the level of an empirical estimate on the student side (Schrader, 1989; see for e.g., Bates and Nettelbeck, 2001). The difference can be expressed as an unstandardized absolute or as a standardized transformed estimate. Only very few studies on this component of judgment accuracy level exist. It is therefore a second research objective of the present paper and operationalized as random intercepts (see for a similar approach for pre-service primary and secondary teachers: Bonefeld et al., 2020).

With respect to the practical implications of these two perspectives for EC education, both relative and absolute accuracies of judgments of teachers can be regarded as important parts of the competence of teachers to adapt their educational activities to the needs of children. Absolute accuracy is needed to evaluate the mean achievement level of a group in relation to a criterion, for example, a curricular objective, or in relation to other groups. This information enables EC teachers to decide about their pacing of educational activities for each group of children (Thiede et al., 2018). Relative accuracy is needed to judge the achievement level of a child in relation to the other children in his/her group. This information enables EC teachers to offer differentiated educational activities to the group by providing individualized feedback and support of knowledge and skill development of children on different levels.

### State of Research: Judgment Accuracy of Teachers in the School Context

Almost all studies on the judgment accuracy of teachers have been carried out in the school context (Hoge and Butcher, 1984; Bates and Nettelbeck, 2001; Kettler and Albers, 2013; Hill and Chin, 2018; Karst et al., 2018). These covered a range of domains, such as mathematics achievement, which is the focus of the present study.

An early systematic review by Hoge and Coladarci (1989) found a medium correlation of *r* = 0.66, with a range of 0.28–0.92, between judgments of teachers and achievement of primary or secondary students (relative accuracy) based on 16 published studies across different domains, including mathematics. Teachers seemed to be more accurate in judging mathematics achievement compared to, for example, achievement in social sciences.

A more recent meta-analysis was carried out by Südkamp et al. (2012), and it included 75 studies carried out in the primary or secondary school since 1989, mostly from the United States. Utilizing (aggregated) correlation coefficients from a multi-level analysis to quantify the relative accuracy of judgments of teachers, Südkamp et al. (2012) found a medium association of *b* = 0.63 that corresponded to a medium correlation of *r* = 0.53 ranging from −0.03 to 0.84. In contrast to Hoge and Coladarci (1989), they did not find differences between judging mathematics achievement and other domains.

In addition to these summaries of domain-specific results, Machts et al. (2016) reported results from 33 studies on relative judgment accuracy in primary or secondary school since 1991, mostly from Germany and the United States, which covered different types of non-domain specific, general cognitive abilities. They found a lower but still medium-sized mean correlation of *r* = 0.43 ranging from −0.18 (as an outlier) to 0.79.

We found considerably fewer studies on *absolute* differences between judgments of teachers and achievement of students in particular and none from the domain of mathematics. Results reported by Bates and Nettelbeck (2001) indicated an overestimation of the reading skills of students. Compared to age-related norms, teachers predicted a score level equal to about 6–12 months ahead of the actual test scores of students. A similar tendency to overestimate students showed up in Freeman's (1993) study of reading judgments. Doherty and Conolly (1985) also found a tendency to overestimate but limited to teachers with less job experience and female teachers if these evaluated girls.

Studies examining judgment accuracy regarding non-cognitive student characteristics, such as motivation, wellbeing, or test anxiety indicated on average a lower correspondence of teacher judgments and student characteristics (e.g., Urhahne and Zhu, 2015). These studies revealed in addition that relative judgments of teachers may not only influence teaching behavior and outcomes of students directly *via* instructional decisions but also indirectly *via* differential expectations toward students with a risk of becoming self-fulfilling prophecies (Urhahne, 2015).

### State of Research: Judgment Accuracy of EC Teachers

The extent to which this state of research can be transferred to EC education is an open question. Evaluating the developmental stage of children in mathematics is a substantial challenge for EC teachers. They have few opportunities to observe each child systematically and repeatedly while working on domain-specific tasks of different levels of difficulty because, in a play-based environment, children may or may not choose activities that can be related to mathematics (Clements and Sarama, 2014). Furthermore, at any given age mathematical skill range of children varies greatly (Resnick, 1989). Children learn number words around the age of 3 years and start to understand more and more precisely the relation between number words and actual quantities. Further on, they are able to recognize the difference between smaller and larger number words and quantities. In addition, they learn to compose and decompose numbers (Fuson, 1988; Krajewski and Schneider, 2009). However, while some children may already be on a level similar to be found in primary schools, others may be behind by 2 or 3 years (Aunola et al., 2004; Aunio and Räsänen, 2016). Such variation may facilitate the judgments of teachers because achievement differences are more salient. However, it is very common at this age that mathematical skills represent a mix of different achievement levels (Krajewski and Schneider, 2009) which in turn may make judgments more difficult for EC teachers.

There are only a few studies available addressing the judgment accuracy of EC teachers. These took place in the slightly more structured environment of preschool. Kilday et al. (2012) applied multi-level modeling to a sample of 318 preschool children in their final year before entering primary school, on average 4.5 years old, and 35 EC teachers. They found associations between judgments of teachers and math skills of children of *r* = 0.54 for the overall score, of *r* = 0.49 for number sense, and of *r* = 0.43 for the subdomains of geometry and measurement. These results mean that teachers rated children who had a test score of 1 SD above or below the mean as being about *half* of an SD above or below the mean. The authors concluded that absolute judgment accuracy of teachers was insufficient for evaluating mathematics achievement in detail. In a follow-up study half a year later, Furnari et al. (2017) were able to replicate the accuracy findings. In addition, they identified several student and teacher characteristics to be associated with the judgment accuracy of teachers, such as the behavior of children or self-efficacy of teachers, thus, biasing the accuracy of their judgments.

Kowalski et al. (2018) reported results from a relative judgment accuracy study with 66 EC teachers and 122 preschool children in their final year before entering primary school (on average 5 years old). The data revealed a correlation of *r* = 0.47 with respect to early math skills and of *r* = 0.60 with respect to reading literacy. Meisels et al. (2001) carried out a study that included a small group of 75 children around 5–6 years old from five preschool classes. Their data revealed a relation of mathematics scores of children regressed on formative assessments of EC teachers, even when demographic background and initial achievement of children were controlled for. Other studies examining relative judgment accuracy regarding early literacy skills indicated medium effect sizes (Cabell et al., 2009; Martin and Shapiro, 2011).

Specifically, with respect to the German context, where our study took place, we could only identify one study that examined the judgment accuracy of EC teachers. Dollinger (2013) carried out a multi-level study with 175 children and 42 teachers about half a year before the end of EC education. During this final year, EC education becomes slightly more structured in Germany than in the purely play-based years before, and teachers have to indicate whether a child is ready to enter primary school at the age of 6 or not. The results revealed a significant relationship between the competence of EC teachers to rank-order children and standardized test results of children in mathematics (*b* = 0.36). However, the relative judgment accuracy of EC teachers was substantially lower than the accuracy of primary teachers who evaluated the same group of children in the same domain during the first year of primary school (*b* = 0.58). The authors indicated that this might be a result of fewer opportunities to evaluate the skills of children in a systematic way, for example, with standardized test instruments, and less mathematics-related training during teacher education.

### Teacher Knowledge as a Predictor of Judgment Accuracy

**Figure 1:**
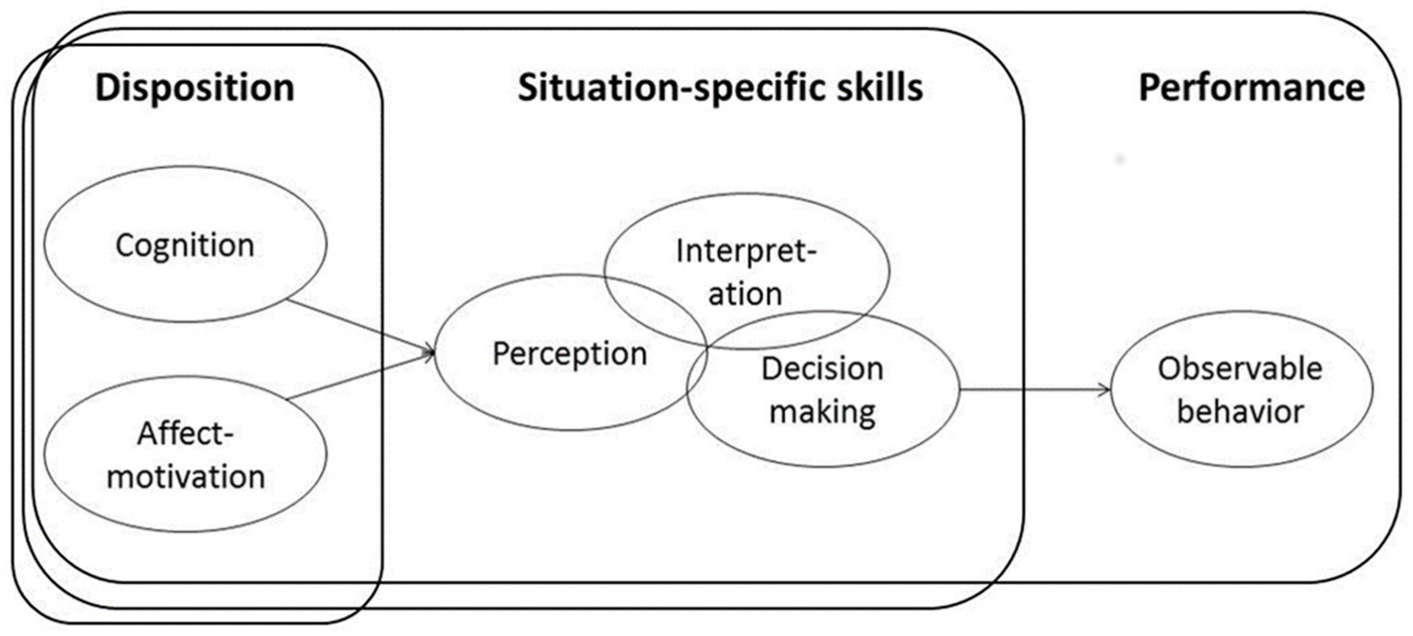
Modeling competence as a continuum (Blömeke et al., 2015a).

Conceptual models of the relationship between knowledge of teachers and their performance in the classroom hypothesize a significant positive relationship (Blömeke et al., 2015a; see Figure 1). In the field of mathematics, relevant facets of teacher knowledge are mathematics content knowledge (MCK), mathematics pedagogical content knowledge (MPCK), and GPK (Shulman, 1986). The cognitive skills of teachers are hypothesized to mediate the relation between dispositional teacher knowledge and their actual performance in class. They are hypothesized to be knowledge-based situation-specific skill facets (Brunner et al., 2013; Hoth et al., 2016). This would mean in our case a positive relation between MCK, MPCK, and GPK and judgment accuracy. The judgment accuracy includes both perception and interpretation but not yet decision-making. Planning of teachers what to do with the information about achievement levels of children and how to adapt their teaching to the needs of children is regarded as a related but separate skill. This skill has recently been addressed in several mathematics-related publications (Vogt et al., 2018; Bruns et al., 2020; Clements et al., 2020).

Based on the model by Blömeke et al. (2015a), Gasteiger and Benz (2018) developed a domain-specific competence model specifically for EC teachers that hypothesizes—with one exception—similar relations. Similar to Blömeke et al. (2015a), they conceptualized judgment accuracy as a situation-specific skill that includes perception and interpretation. Moreover, the skill was similarly conceptualized as knowledge based on mathematics content and pedagogical content knowledge as relevant knowledge facets. The difference to Blömeke et al. (2015a) is that GPK was not included in the model.

The hypothesized relations could repeatedly be supported by data with respect to a broad range of knowledge, including GPK, cognitive skills, and performance facets for both primary and secondary school teachers (Baumert et al., 2010; Nehls et al., 2020). Specifically, with respect to judgment accuracy, Hill and Chin (2018) were able to provide evidence for a positive relation between MCK and their accuracy with respect to mathematics achievement of secondary school students. Glogger-Frey et al. (2018) found a corresponding positive relation with respect to the GPK of pre-service secondary teachers and their judgment accuracy. Studies by Lorenz and Artelt (2009) revealed that judgment accuracy measures correlated significantly within domains but not across domains. This result may be interpreted as indirect evidence for the relevance of MCK and/or MPCK. Similarly, intervention studies by Thiede et al. (2015) with primary school teachers showed that content-related professional development courses, supposed to increase teachers' content and pedagogical content knowledge, improved teachers' content-related judgment accuracy.

In contrast to a conceptualization of judgment accuracy as a knowledge-based performance facet, Rausch et al. (2015) did not find systematic relations between the content of secondary teachers and pedagogical content knowledge of German and their accuracy to evaluate text comprehension of their students. Binder et al. (2018) were also unable to provide systematic evidence for a relation between judgment accuracy of secondary school teachers in the field of mathematics and their content-related knowledge. However, knowledge and accuracy differences between the teacher groups examined (teaching in academic tracks and having had a longer teacher education program vs. teaching in non-academic track and a shorter teacher education program) may be regarded as indirect evidence for such a relation.

Overall, the state-of-research is inconclusive with respect to primary and secondary teachers while we were not able to identify any studies that examined the relation of judgment accuracy of EC teachers to their knowledge facets.

## Research Questions

The first purpose of this study was to examine the correspondence between relative and absolute judgments of EC teachers and the mathematical skills of children. The two components of accuracy are relevant with respect to planning educational activities for groups of children (absolute accuracy) and to the individual support of the mathematical development of children (relative accuracy).

Based on the state of research on *relative accuracy*, we assumed a positive relation of medium effect size between EC teachers' judgment of the mathematical skills of children and these children's skills, identified via an estimation of random slopes (Research Question (RQ) 1a). That is, with respect to the rank component of judgment accuracy, we assumed that children with higher scores on a well-established standardized test of their mathematical skills (“Mathematische Basiskompetenzen im Kindergartenalter,” MBK-0; Krajewski, 2018) also showed higher scores on a well-established tool used by teachers to evaluate skills of children (“Kinder Diagnose Tool,” KiDiT; Walter-Laager et al., 2011; for details about both instruments and the model estimated, refer to the Methods section below).

With respect to *absolute judgment accuracy*, we assumed differences between EC teachers' evaluation of children's mathematical skills and their actual skills as identified in the estimation of random intercepts (RQ 1b). That is, the level of the KiDiT score predicted from the MBK-0 score would not necessarily correspond to the actual level of the KiDiT score. We are not able to formulate a directional hypothesis given the inclusive state of research which states either a tendency to overestimate skills of children (Bates and Nettelbeck, 2001) or a tendency to underestimate them (MacDonald and Murphy, 2019).

The second purpose of this study was to identify *predictors* of judgment accuracy of EC teachers. There are no studies from EC education available in this respect, and the state of research with respect to primary and secondary teachers is inconclusive, making this part of our study is exploratory. Conceptual models (see Blömeke et al., 2015a) point to a potential moderating effect of domain-specific MCK and MPCK of EC teachers on the relationship between judgments of teachers and mathematical skills of children while the role of non-domain specific GPK is more uncertain (RQ 2).

## Methods

### Sample

Early childhood teachers and children were recruited by contacting all EC institutions in Berlin and Brandenburg *via* email and asking for voluntary participation. Teachers and parents of children had to agree and did therefore not constitute representative samples. Children were assessed at various measurement occasions by means of the MBK-0 test (two measurement occasions) and the KiDiT evaluation tool (five measurement occasions). For the current analysis, we used data from the first measurement occasion (*t1*) only because at *t2*, less data on the children's level are available due to attrition. In addition, the RQs are focused on cross-sectional analysis. Extending the scope of interest to a longitudinal setting, for instance, the stability of judgment accuracy would require further theoretical and methodological considerations.

Overall, data from 350 children are available in the KiDiT dataset at *t1* and from 337 children in the MBK-0 dataset at *t1*. To arrive at a final dataset for the analysis, we merged the two datasets by the variable “child” and checked whether the teachers to which the children were assigned were identical across the two datasets. We selected only children for which the teacher information was congruent and arrived at a dataset with 268 children and 39 teachers, respectively. The children were on average 4.46 years old (SD = 0.85; min–max = 2.70–6.64 years; for four children, age information was missing). The range of the average group age varied from 3 to 5.87 years. For one group, age information was not available. Slightly more than half of the children were girls (54%), and 46% of the children were boys. For 21 children, the gender information was missing. Group size varied between 3 and 10 children and was on average 7 children per group.

The EC teachers were career starters with a working experience of up to 5 years and were tested on their MCK, MPCK, and GPK online (data on all three knowledge facets were available for 34 of the 39 participating EC teachers). They were on average 33 years old (SD = 9.39; min–max = 22–57; for 9 of the 39 teachers, age information was not available). Regarding the highest educational degrees that were achieved by the teachers, 6 EC teachers had a “Realschulabschluss” (school leaving certificate of secondary school), 7 teachers had the “Fachhochschulreife” (college entrance certificate), 14 teachers have achieved the “Abitur” (university entrance certificate), and 3 teachers have achieved an “abgeschlossenes Hochschulstudium” (college degree). This heterogeneity reflects the heterogeneity of qualifications of EC teachers in Germany well. For nine teachers, the information was not available.

### Instruments

Judgments of teachers about the mathematical skills of children stem from the “KiDiT” meant to document the development of children in several domains, among others mathematics (Pfiffner and Walter-Laager, 2009; Walter-Laager et al., 2011). The tool is well-established in German-speaking countries, in particular, in Switzerland, and is recommended for evaluating the development age of children aged 0.5–8 years in Zurich. The standardized part of the instrument used in the present study includes 25 items related to mathematics. These have to be rated on 5-point Likert scales from “Does not apply” to “Does apply.” The items cover three mathematical domains: number (e.g., “The child is able to name precursors and successors of a number.”), quantity (e.g., “The child can compare simple quantities.”) and geometry (e.g., “The child identifies shapes of figures and forms, e.g., triangle, circle, square, rectangle, cube, and sphere, in the environment and on illustrations”).

To explore the dimensionality of the KiDiT instrument, we conducted an exploratory factor analysis for categorical data using the Software Mplus 8.3 (Muthén and Muthén, 1998–2017) based on all available KiDiT data at *t1* (*N* = 350). Examining the first eigenvalues of the sample correlation matrix (12.281, 1.924, 1.604, …) suggests that the KiDiT ratings are unidimensional. To assess the reliability of the instrument, we conducted an analysis with the generalized partial credit model (Muraki, 1992) as implemented in the R-package “mirt” (Chalmers, 2012). The estimated empirical reliability was 0.94. In the analysis dataset with 268 children, the mean KiDiT score could range from 0 to 4, the average rating was *M* = 2.44 (SD = 0.87; for five children KiDiT ratings were missing).

The children's mathematical skills were assessed with a standardized and well-established test targeting children aged 3 to 6 years (MBK-0; Seeger et al., 2014; Krajewski, 2018). MBK-0 is a screening tool for several basic skills that are part of the national curriculum for EC education in Germany, among others numerical skills [Jugendministerkonferenz (JMK) and Kultusministerkonferenz (KMK) Deutschland, 2004]. It has been used regularly in EC research and meets psychometric quality criteria, such as objectivity, reliability, and validity, in particular, prognostic validity of developmental risks. Beginning with the age of 3 years up to 6.5 years, normed scores and thresholds for developmental risks are available for each half-year (Krajewski and Ennemoser, 2013).

The MBK-0 instrument includes nine tasks from number and quantity. Five tasks are targeting 3-year-olds who have to count forward and backward, identify subsequently and preceding numbers, and read numbers up to 20. Three tasks are targeting 4-year-olds who have to compare, order, and assign numbers. One task is targeting children up to 6.5 years who have to compare quantities. Children were tested individually and needed up to 15 min to complete the screening depending on their age.

To assess the reliability of the MBK-0, we used all the information available at *t1* (*N* = 337) and conducted a confirmatory factor analysis for mixed response format in Mplus 8.3. Reliability was estimated by dividing the variance of the estimated factor scores by the sum of this variance and the average error variance. The estimated reliability was at 0.92. We used the sum score on the MBK-0 as an indicator for the development of a child's state in mathematics. The maximum score was 44 points. Our sample (*N* = 268) achieved on average 14.64 points (SD = 11.96; for 16 children the MBK-0 score was missing). The fact that 14.64 points can be considered relatively low is because the younger children have achieved relatively low scores (see Supplementary Figure SI 1). This may be partly due to the scoring scheme as given in the MBK-0 manual. Very young children are presented with the first items on the MBK-0 only to avoid a cognitive overload. It is assumed that younger children, due to their developmental level, are not able to solve the more difficult items.

Knowledge of EC teachers was assessed in three domains with instruments validated in a range of other studies (Blömeke et al., 2017). Example items can be found in Supplementary Material SI 2. The assessment of GPK consisted of 30 multiple-choice or bundled items or items requiring open responses. These covered general foundations from educational theory, psychology, and instructional research. The MPCK assessment consisted of 36 items in a multiple-choice, bundled, or open-response format. These content-wise items covered diagnosing the developmental state of children in mathematics and designing an informal learning environment that fosters the mathematical learning of children between the ages of 3 and 6. The assessment of MCK consisted of 23 multiple-choice or open-response items. These covered numbers and operations, geometry, quantity and measurement, data, combinatorics, and chance. The tests cover EC-specific knowledge beyond general cognitive abilities (Jenßen et al., 2019).

To assess the reliability of these instruments, we conducted item response analyses using the 2-parameter-logistic-(2PL) model as implemented in the R-package “mirt” (Chalmers, 2012) based on all available teachers in the dataset. This dataset also contains information about ca. 120 teachers who did not partake in the current study. The reason for including all available information on the teacher level was that a sample of 39 teachers could be considered too small for reliability estimation. In addition, Blömeke et al. (2015b) have already reported reliabilities for the knowledge tests, which were 0.68 (GPK), 0.87 (MPCK), and 0.88 (MCK). Here, we were interested in the reliability estimates for the current sample of teachers.

Visual inspection of the score histograms indicated that some EC teachers achieved 0 points on the tests, while otherwise, the scores showed approximately a normal distribution. The scores of EC teachers with 0 points were set to missing because they might indicate that the respective teachers did not work appropriately on the online tests. The estimated empirical reliabilities were 0.80 (GPK, *N* = 166), 0.81 (MPCK, *N* = 156), and 0.86 (MCK, *N* = 152). This complements earlier findings by Blömeke et al. (2015b), who reported the reliabilities as 0.68 (GPK), 0.87 (MPCK), and 0.88 (MCK).

We included the sum scores of these three EC teacher knowledge domains in our models. The average sum score on the MCK test for our sample (*N* = 39) was 13.04 points (SD = 6.28, min = 1; max = 22; MCK scores of 13 EC teachers were missing). The average MPCK score was 20.41 points (SD = 5.11; min = 10; max = 30; MPCK scores of 12 EC teachers were missing). The mean score on the GPK test was 14.93 points (SD = 5.38, min = 1, max = 24; GPK scores of 10 EC teachers were missing).

### Data Analysis

Since children were nested within EC teachers, we applied multi-level modeling (Bryk and Raudenbush, 1992; Hox, 2010; Snijders and Bosker, 2011) to examine our RQs. The modeling approach is similar to the approach presented by Dollinger (2013). In addition, we used a Bayesian estimation procedure as implemented in Mplus 8.3 (Muthén and Muthén, 1998–2017) that provides within-level effect size indices averaged across teachers and on the between-teacher levels.

#### Model 1

Research questions 1a and 1b were tested by modeling the relationship between the judgment of teachers and mathematical skills of children on the between- (level-2) and within-levels (level-1).

The level-1 equation is as follows:


(1) $$\begin{array}{l} y_{ij}=\beta_{0j}+\beta_{1j}x_{1ij}+\varepsilon_{ij}. \end{array}$$

Here, *y*_*ij*_ is the *z*-standardized mean KiDiT score of child *i* nested within teacher *j*, *x*_1*ij*_ is the *z*-standardized MBK-0 score of child *i* nested within teacher *j*, and ε_*ij*_ is a level-1 residual. The mean KiDiT scores and the MBK-0 scores were *z*-standardized to give the random intercepts an interpretable meaning. Thus, β_0*j*_ is the expected KiDiT score of a child with an average MBK-0 score in cluster *j*. An estimated coefficient of 0 would indicate that a child that has achieved an average MBK-0 score would be expected to achieve an average KiDiT score based on the rating of teacher *j*. A value greater than zero would indicate an overestimation and a value smaller than zero would indicate an underestimation of the KiDiT score of such a child relative to the objective MBK-0 test score. In other words, the random intercepts are indicative of the level component of the judgment accuracy of a teacher (*absolute* accuracy). The coefficient β_1*j*_ is indicative of the within-cluster relationship between the KiDiT and MBK-0 scores of children. High positive coefficients would indicate a positive relationship between MBK-0 test scores and KiDIT ratings within clusters and are indicative of the rank component of judgment accuracy of a teacher (*relative* accuracy). The level 2 equations are as follows:


(2) $$\begin{array}{l} \beta_{0j}=\gamma_{00}+u_{0j} \end{array}$$

(3) $$\begin{array}{l} \beta_{1j}=\gamma_{10}+u_{1j} \end{array}$$

The coefficient γ_00_ is the expected average intercept and γ_10_ is the expected average slope. The coefficients *u*_0*j*_ and *u*_1*j*_ are level-2 residuals for teacher *j*. Regarding the level-2 residuals, we adopted the usual assumption of multilevel modeling, that is, multivariate normal distribution of the random coefficients with zero means and normal distribution of the level-1 residuals with a mean of zero. We checked the level-1 residuals visually for normality with the R-package lme4 (Bates et al., 2015). The empirical residual distribution looked normal with an expectation of close to zero.

#### Model 2

For examining RQ 2, we extended Model 1 by including the MCK, MPCK, and GPK scores of teachers into the analysis, resulting in a slope-and-intercepts-as-outcomes-model. The aim was to assess, whether the rank- and the level component of the judgments of teachers could be explained by teacher characteristics.

The level-2 equations are:


(4) $$\begin{array}{l} \beta_{0j}=\gamma_{00}+\gamma_{01}x_{1j}+\gamma_{02}x_{2j}+\gamma_{03}x_{3j}+u_{0j} \end{array}$$

(5) $$\begin{array}{l} \beta_{1j}=\gamma_{10}+\gamma_{11}x_{1j}+\gamma_{12}x_{2j}+\gamma_{13}x_{3j}+u_{1j} \end{array}$$

In these equations, *x*_1*j*_, *x*_2*j*_, and *x*_3*j*_ are the MCK, MPCK, and GPK scores of teachers *j*. These scores were centered to give the coefficients γ_00_ and γ_10_ an interpretable meaning. The coefficient γ_00_ is the expected level component for a teacher with average test scores, and γ_10_ is the expected slope component for a teacher with average test scores. Coefficients γ_01_, γ_02_, and γ_03_ capture the relationship between the level components and teacher test scores and the coefficients γ_11_, γ_12_, and γ_13_ are indicative of the relationship between the rank component and the teacher test scores. The coefficients *u*_0*j*_ and *u*_1*j*_ are level-2 residuals of teacher *j*. In accord with standard practice in multilevel modeling, a multivariate normal distribution with means of zero is assumed for the level-2 residuals.

#### Model Estimation

The models were estimated using a Bayesian estimation approach and the Gibbs algorithm for Markov chain Monte Carlo as implemented in Mplus 8.3. (Muthén and Muthén, 1998–2017). Uninformative priors, two processors, and two chains were used. The reason for using uninformative priors was that we did not want to introduce any prior assumptions about the model parameters into the analysis. A thinning value of 50 was utilized, that is, only every 20th sample from the posteriors was used to account for possible autocorrelations in the Markov chains. The convergence criterion was set in such a way that at least 5,000 samples were collected per chain and the potential scale reduction factor had to be smaller than 1.01.

The autocorrelation plots and the chains were checked visually for small autocorrelations and convergence, which gave satisfactory results. The posterior median of the parameters was used as a point estimate, and the quantiles of the posterior draws were used to construct 95% credibility intervals (CI) for the parameter estimates. On the within-level, standardized estimates averaged over clusters are reported (Schuurman et al., 2016). Thus, the individual effects on the teacher level are standardized on the within-teacher variance. Standardized effects are available for each teacher, which are averaged to assess the central tendency of the individual effects. To account for missing data, we included the variance of the MBK-0 scores into the analysis and modeled the covariance between the teacher-level variables. Thus, all available information was used.

Given the number of missing values, we cross-checked the robustness of the results based on a full Bayesian analysis. We conducted an additional analysis using multiple imputations in Mplus. To account for missing data, we imputed 20 datasets based on the unrestricted H1 model. Maximum likelihood estimation with robust standard errors (MLR) was used, and the results were aggregated in Mplus. The results were comparable to the full Bayesian analysis. We chose to report the Bayesian analysis here, as this estimation method has the additional benefit, that standardized effects averaged across teachers are available.

More details, such as the data, Mplus scripts, and outputs, can be found in the Supplementary Material.

## Results

### Absolute and Relative Judgment Accuracies of EC Teachers (RQs 1a and 1b)

**Figure 2:**
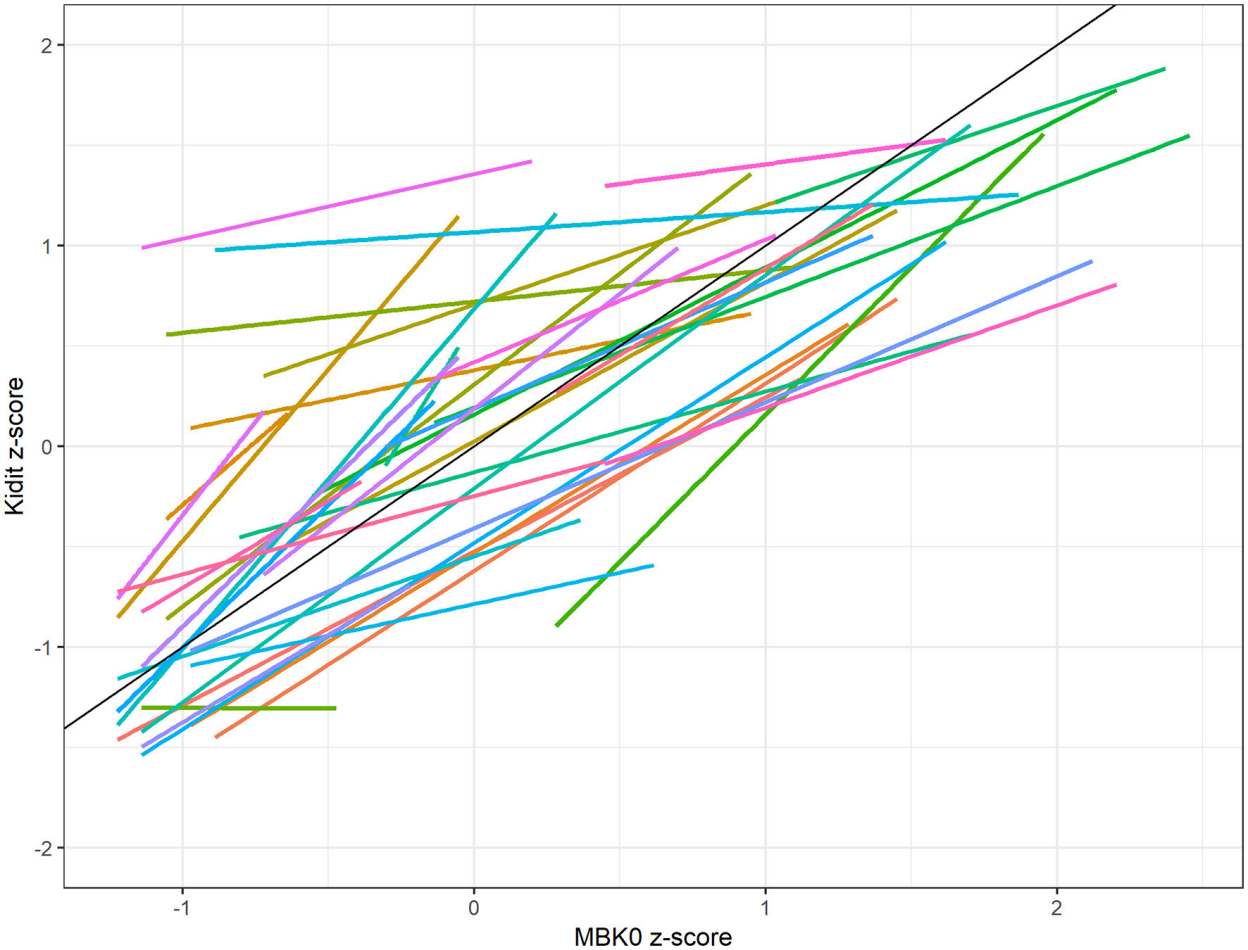
Teacher-specific regression lines (teacher judgment characteristic curves) of KiDiT *z*-scores of children regressed on MBK-0 *z*-scores of children. The slim diagonal line with slope 1 and intercept 0 across the plot represents an ideal judgment behavior that is perfectly in accord with the MBK-0 scores. KiDiT, Kinder Diagnose Tool; MBK-0. Mathematische Basiskompetenzen im Kindesalter.

On a descriptive level, Figure 2 shows teacher-specific regression lines of their children's *z*-standardized mean KiDiT scores on their children's *z*-standardized MBK-0 scores. The plot suggests that the MBK-0 scores are positively related to the KiDiT ratings of teachers. In general, teachers seem to do relatively well in to ranking their children with regard to their mathematical skills as assessed by the MBK-0 test. There seems to be some variation in the degree of relative teacher judgment accuracy and a few cases with weaker accuracy but no cases where a teacher rating is completely off compared to the MBK-0 score.

The intercepts indicate to what extent teachers would overestimate or underestimate a child with the average MBK-0 score and refer to the absolute judgment accuracy of teachers (see Figure 2). It is important to note that a positive or negative intercept represents a local tendency to respectively over- or under-estimate a child with the average MBK-0 score. This interpretation does not necessarily extend across the whole range of MBK-0 scores, as the slopes of the regression lines can vary. An analogy may help to understand the meaning of these regression lines further: when the teachers are assumed to be test items and when the MBK-0 scores are thought of as values on a latent dimension representing a skill, then the regression lines are linear item response curves, where the intercepts represent the item easiness and the slopes represent the discrimination parameters. An ideal judgment behavior relative to the MBK-0 is represented by an intercept of 0 (average easiness) and a discrimination parameter of 1. In this sense, the regression lines represent teacher judgment characteristic curves relative to the MBK-0. The variation of the intercepts suggests that interindividual differences in the level component of teacher judgment accuracy (absolute accuracy) exist.

To statistically substantiate these observations, we applied model 1 to the data. Table 1 presents the results. The intercept is γ_00_ = 0.037 [95% CI: (−0.161; 0.233)] which suggests that the teachers are—on average—able to correctly judge the developmental level of a child in mathematics with an average MBK-0 score. However, a “non-significant” result where the 95% CI contains the value of zero is not “proof” for the null hypothesis that the mean intercept is exactly zero. The intercept variance [$\sigma_{u_{0j}}^{2}$ = 0.304; 95% CI: (0.175; 0.554)] suggests that teachers vary significantly regarding their absolute judgment accuracy.

**Table 1:** Model 1: KiDiT scores of children multilevel-regressed on MBK-0 scores of children.

**Coefficient**	**Label**	**Estimate**	**95%-CI**	**Standardized estimate**	**95%-CI**
Level-1 residual variance					
$\sigma_{\varepsilon_{ij}}^{2}$		0.221^*^	[0.181; 0.273]	–	–
Random effects					
$\sigma_{u_{0j}}^{2}$	Intercept variance	0.304^*^	[0.175; 0.554]	1.000	–
$\sigma_{u_{1j}}^{2}$	Slope variance	0.083^*^	[0.029; 0.203]	1.000	–
*cov*[*u*_0*j*_, *u*_1*j*_];		−0.061	[−0.165; 0.034]	−0.423	[−0.831; 0.177]
Fixed effects					
γ_00_	Intercept	0.037	[−0.161; 0.233]	–	–
γ_01_	Slope [MBK-0]	0.724^*^	[0.590; 0.856]	–	–
Within-level standardized effects averaged across teachers					
	KiDiT on MBK-0	–	–	0.794^*^	[0.718; 0.849]
	Residual variance	–	–	0.347^*^	[0.271; 0.438]

* *The 95% credibility interval (CI) does not include the value of 0. Deviance Information Criterion (DIC) = 1118.97. Average $R_{within}^{2}$ = 0.653 (95% CI: [0.561; 0.729]). n_teacher_ = 39; n_children_ = 268. Standardized estimates are given where appropriate. The within-level standardized effect of the regression of the KiDiT scores on the MBK-0 scores within one teacher's group and the residual variance are averaged over clusters (teachers). KiDiT, Kinder Diagnose Tool*.

The average slope is γ_01_ = 0.724 [95% CI: (0.590; 0.856)]. This indicates that for a typical teacher, two children differing by 1 SD in their MBK-0 scores are expected to be 0.742 SDs apart on their KiDiT scores. The within-level standardized effect averaged across all teachers, which can be interpreted as an average correlation coefficient, is 0.794. On average, across teachers, 65.3% of the variation in the KiDiT scores is explained by the MBK-0 scores of children (95% CI: [0.561; 0.729]). This effect size can be regarded as high given Cohen (1969) classification of effect sizes in multiple regression. The result suggests that teachers are—on average—able to rank the children well with regards to their mathematical skills by means of the KiDiT instrument and indicates a high degree of relative judgment accuracy. The estimated slope variance is $\sigma_{u_{1j}}^{2}=$ 0.083. The 95% CI here is (0.029; 0.203) and does not include the value of 0.

### Moderation of Judgment Accuracy of Teachers (RQ 2)

Table 2 shows the results based on model 2 that whether knowledge of teachers in three dimensions (MCK, MPCK, and GPK) moderated the within-group relation between KiDiT and MBK-0 (RQ 2). Similar to model 1, the expected average intercept is γ_00_ = 0.038 [95% CI: (−0.169: 0.251)] and the expected average slope is γ_10_ = 0.742 [95% CI: (0.592; 0.902)]. In line with our assumption, the GPK of EC teachers does not explain variation in judgment accuracy (see γ_03_ and γ_13_ in Table 2). However, in contrast to our assumptions, none of the content-related teacher-level predictors explains variation in the absolute judgment accuracy of teachers (intercepts; see γ_01_ and γ_02_ in Table 2) or their relative judgment accuracy (slopes; see γ_11_ and γ_12_), either. On the between-teacher level, correlations of knowledge scores of teachers are *r* = 0.810 [95% CI: (0.526; 0.925)] for MCK and MPCK, *r* = 0.729 [95% CI: (0.346; 0.889)] for MCK and GPK, and *r* = 0.737 [95% CI: (0.393; 0.888)] for MPCK and GPK which has to be regarded as high.

**Table 2:** Model 2: Predicting the random intercepts (absolute judgment accuracy, level component) and random slopes (relative judgment accuracy, rank component) by test scores of teachers.

**Coefficient**	**Label**	**Estimate**	**95%-CI**	**Standardized estimate**	**95%-CI**
Level-1 residual variance					
$\sigma_{\varepsilon_{ij}}^{2}$		0.218^*^	[0.179 0.270]		
Random-effects					
Variances and covariances					
$\sigma_{u_{0j}}^{2}$		0.319^*^	[0.172; 0.619]		
$\sigma_{u_{1j}}^{2}$		0.102^*^	[0.032; 0.250]		
*cov*[*u*_0*j*_, *u*_1*j*_]		−0.061	[−0.182; 0.051]	−0.367	[−0.825; 0.244]
Fixed effects					
γ_00_	Intercept	0.038	[−0.169; 0.251]	0.060	[−0.263; 0.389]
γ_01_	MCK	0.029	[−0.031; 0.089]	0.385	[−0.383; 1.131]
γ_02_	MPCK	−0.024	[−0.098; 0.046]	−0.258	[−1.030; 0.467]
γ_03_	GPK	−0.028	[−0.092; 0.035]	−0.276	[−0.849; 0.354]
γ_10_	Slope [MBK-0]	0.742^*^	[0.592; 0.902]	2.025^*^	[1.225; 3.442]
γ_11_	MCK	−0.002	[−0.040; 0.040]	−0.046	[−1.009; 0.769]
γ_12_	MPCK	0.016	[−0.032; 0.062]	0.301	[−0.571; 1.109]
γ_13_	GPK	−0.021	[−0.066; 0.027]	−0.362	[−1.036; 0.446]
Covariance/correlation of teachers' test scores on level-2					
*cov*[*MCK, MPCK*]	MCK with MPCK	43.311^*^	[19.755; 95.513]	0.810^*^	[0.526; 0.925]
*cov*[*MCK, GPK*]	MCK with GPK	36.114^*^	[12.990; 79.679]	0.729^*^	[0.346; 0.889]
*cov*[*MPCK, GPK*]	MPCK with GPK	29.746^*^	[11.927; 64.146]	0.737^*^	[0.393; 0.888]
Within-level standardized effects across teachers					
	MBK-0 on KiDiT	–	–	0.785	[0.698; 0.845]
	Residual variances	–	–	0.350	[0.274; 0.440]

* *The 95%credibility interval (CI) does not contain the value of 0. Deviance Information Criterion (DIC) = 1578.49; Average $R_{within}^{2}$= 0.650 (95% CI: [0.560, 0.726]) $R_{\beta_{0j\left(between\right)}}^{2}$ = 0.171 [95% CI: (0.019, 0.467)]; $R_{\beta_{1j\left(between\right)}}^{2}$ = 0.217 [95% CI: (0.020, 0.581)]; n_teacher_ = 39; n_children_ = 268. Standardized estimates are given where appropriate. The standardized within-level effects and residual variances are averaged over clusters (teachers). MCK, mathematical content knowledge; MPCK, mathematics pedagogical content knowledge; GPK, general pedagogical knowledge; KiDiT, Kinder Diagnose Tool*.

As was the case for model 1, on average 65.0% of the variation in the KiDiT scores is explained by MBK-0 scores of children [95% CI: (0.560, 0.726)]. Only 17.1% in the variation of the intercepts (level component or absolute accuracy) is explained by knowledge scores of teachers [95% CI: (0.019, 0.467)], and just 21.7% of the variation in the slopes (rank component or relative accuracy) is explained by teacher knowledge [95% CI: (0.020; 0.581)].

Overall, the results suggest, that EC teachers are relatively well able to judge the mathematical skills of the children within their group relative to each other and with respect to their absolute level. However, content-related knowledge scores of teachers explained unexpectedly only little variance in the variation of the rank and level components. All 95% CIs of the parameters that represent the effects of teacher characteristics on the judgments' rank and level components included the value of zero.

### Stability Analysis

To cross-check the stability of the results, we conducted a complementary analysis in which we have excluded the items for assessing the geometry domain from the KiDiT. The reason for this was that geometry is not assessed in the MBK-0. The results are reported in Supplementary Material SI 3 and are virtually identical to the results reported here. This was to be expected, as the exploratory factor analysis suggests that the KiDiT items are empirically relatively unidimensional, albeit measuring different domains on the content level.

## Summary and Discussion

Two research objectives shaped our study: we wanted to examine whether EC teachers are able to accurately diagnose the mathematical skills of children in a play-based kindergarten environment where educational activities are not extensively pre-planned or implemented in a systematic way, but where children's play is used as a starting point for providing educational activities. This first research objective had two dimensions: (a) accurately judging the relative standing of children with regard to their skills within an EC teacher's group and (b) accurately judging the *absolute* level of these skills. In both cases, an objectively measured test score was used as the criterion for estimating the accuracy of teacher ratings.

The second objective of this study was to identify predictors of judgment accuracy of EC teachers. Based on conceptual models of teacher competence, we assumed that their content-related dispositional knowledge facets in terms of MCK and MPCK would play a role in their accuracy as a situation-specific skill while the role of GPK was more uncertain.

To the best of our knowledge, our study was the first one applying standardized testing of EC teachers, so that robust indicators of their knowledge have so far been lacking. Moreover, almost all research was done on judgment accuracy of primary and secondary school teachers who diagnose student achievement in the structured context of classrooms with many opportunities of observing students while they work on mathematical tasks carefully pre-designed. The few EC studies available took place in preschool environments that prepare for primary school and are therefore often slightly more structured and pre-planned than fully play-based EC environments.

### Absolute and Relative Judgment Accuracies of EC Teachers (RQs 1a and 1b)

Regarding our first research question (RQ 1a), the data revealed that EC teachers are able to accurately rank the children within their group with regard to their mathematical skills as assessed by the MBK-0 test. There was only little variation in the degree of relative teacher judgment accuracy, which indicates that this task is mastered by almost all teachers. EC teachers are mostly able to correctly identify the differences in mathematics achievement among the children in their group, which means that a child with a higher MBK-0 score can expect to get a better KiDiT rating. This is an important finding since such relative judgment accuracy provides EC teachers with the information needed to differentiate their educational activities and to provide individualized feedback and support of all children, no matter the developmental level they are at.

Considering that on average two-thirds of the variation in the KiDiT scores are explained by the MBK-0 scores of children, which corresponds to an average correlation of about 0.80, the relative judgment accuracy of EC teachers in our study is at least similar if not higher than the accuracy reported in the literature for primary and secondary teachers (see in particular the systematic reviews and meta-analysis by Hoge and Coladarci, 1989; Südkamp et al., 2012; Machts et al., 2016). Given that our study took place in an unstructured play-based EC environment without systematic and frequent formative or summative assessments that characterize schooling, this is a remarkable result. It is much harder for an EC teacher to judge the achievement level of children given that activities or statements related to mathematics are more implicit and rare.

While relative accuracy is needed for differentiated educational activities, absolute teacher judgment accuracy is needed for decisions about the pacing of these activities on the group level, for example, to meet a curricular goal (RQ 1b). In this respect, our data revealed that EC teachers are on average also able to correctly judge the mathematical level as revealed by the MBK-0 score. However, absolute accuracy varied significantly by EC teacher. In practice, accuracy will therefore depend heavily on exactly which EC teacher is making the judgment.

Nevertheless, the average correspondence of judgments of teachers and test scores is a surprising result and deviates from studies examining absolute accuracy in the contexts of preschool. Here, either an overestimation of skills of children was found that equaled to be about 6–12 months ahead of the actual test scores (for a similar tendency see Freeman, 1993; Bates and Nettelbeck, 2001) or an underestimation (MacDonald and Murphy, 2019). It might be that the larger variation in mathematical skills of children in the German context of heterogeneous groups ranging from 3 to 5 or 6 years and the larger variety of mathematical skills at the lower age in general (Aunola et al., 2004) facilitate the judgments of EC teachers because skill differences are more salient.

Given the generally limited training in making such judgments provided to EC teachers during teacher education (Blömeke et al., 2017; Gasteiger et al., 2021), these are promising results. The literature about adaptive teaching is clear that EC teachers' planning of educational activities is dependent on accurate information about the achievement levels of children (Vogt et al., 2018; Bruns et al., 2020; Clements et al., 2020). Only then they are able to adapt their teaching to the needs of children (Wullschleger, 2017; Meier-Wyder, 2020).

### Moderation of Judgment Accuracy of Teachers (RQ 2)

The objective of our second RQ was to dig deeper into the potential characteristics of EC teachers that could predict their judgment accuracy. Models of teacher competence (Blömeke et al., 2015a; Gasteiger and Benz, 2018) conceptualized judgment accuracy as a knowledge-based situation-specific skill, such as perception and interpretation.

The data revealed that neither GPK nor MCK or MPCK moderated the within-group relationship between KiDiT ratings and MBK-0 scores. In particular, the lack of domain-specific knowledge effects is an unexpected result. Thus, we have not been able to identify any knowledge facet that predicts relative or absolute judgment accuracy. In that respect, our results are in line with the previous studies using EC teacher education degrees or course credits which neither had any effects (Lin and Magnuson, 2018). However, the result is against our assumptions since we expected standardized tests of teacher knowledge that would provide teacher covariates proximal enough to be related to judgment accuracy.

It remains thus an open question which characteristics of EC teachers facilitate their judgment accuracy. There are several potential interpretations of this result. It could, firstly, point to a *conceptual* challenge. The models by Blömeke et al. (2015a) and Gasteiger and Benz (2018) may be underspecified in that they do not include sufficiently other characteristics that are relevant and influence the relation between knowledge and judgment accuracy of EC teachers. Although hard to imagine, an alternative version of this conceptual interpretation would be that knowledge of EC teachers simply may be less relevant for their judgment accuracy than conceptualized – both with respect to domain-specific MCK and MPCK and with respect to the domain-general GPK.

The second interpretation of this result could point to a potential *validity* challenge of the assessments applied. All instruments have been validated in separate studies for different purposes, including the assessments of knowledge of EC teachers. However, it might be that the instruments which cover MCK, MPCK, and GPK in broad ways are not specific enough to assess exactly those knowledge facets relevant for judgment accuracy of teachers [as Gasteiger and Benz (2018) suggest, for example]. Very specific knowledge about developmental stages of mathematics achievement, for example, could be a crucial facet but are represented by a few items only in the assessment. In a general sense, Depaepe et al. (2013) point in addition to the challenge that one may need to assess knowledge differently when the purpose is to relate it to constructs that are situation-specific and thus vary across situations.

## Limitations

Before we turn to conclusions, we need to point out the limitations of our study. The first one is related to the sample, which is not representative. Though all EC institutions in Berlin and Brandenburg were contacted *via* E-Mail, participation in the study was voluntary for the teachers and parents of the children. The second limitation is related to the domain of our study. We focused on mathematics, which means that interpretations have to be restricted to this domain. Given the diversity of results across domains with respect to primary and secondary school teachers and given that these studies did not find substantial correlations of judgment accuracy across domains (Spinath, 2005; Binder et al., 2018), the accuracy of EC teachers may neither be a general but a domain-specific characteristic. Third, we have no concise information for how long the individual teachers knew their children in their groups. It could be speculated that the teachers have known the children in their group since the age of 3, as children in Germany are assigned to a new group starting from that age. However, it would be advisable to collect this information in future studies, because the duration of acquaintance may influence the judgment accuracy.

Furthermore, although we could utilize a sample size sufficient for multi-level modeling and in line with other studies on teacher accuracy, the number of predictors that could be included on the between level was limited for reasons of statistical power. Therefore, results have to be interpreted with care. A study with a larger sample size on the teacher level could provide more robust evidence with respect to potential predictors of judgment accuracy of teachers.

## Conclusions and Implications for Future Research

There is little research about play-based EC education applying standardized testing of children so that applying a well-established instrument such as the MBK-0 can be regarded as a specific strength of our study. Evaluating skills of children in a standardized way is—with the exception of intelligence tests for the identification of intellectual disabilities or giftedness (Kranzler et al., 2016)—similarly rare so that the assessment of judgment accuracy of teachers with the help of the standardized KiDiT tool can be regarded as another strength. Finally, we are not aware of any study that has tested knowledge of EC teachers in a standardized way so that our results go far beyond the current state of research also in this respect.

Our main findings are that within EC teachers' groups of children, a high degree of *relative* judgment accuracy and in addition on average a decent degree of *absolute* judgment, accuracy exists. These results provide also further evidence for the validity of the KiDiT tool which is widely used in German-speaking countries to rate the mathematical skills of children. Our results can be regarded as quite robust due to using standardized measures. Inferences drawn based on such measures reveal typically stronger validity than on unstandardized measures (Meehl, 1954; Grove and Meehl, 1996). We suggest that researchers increase their efforts to implement standardized tools also in the context of EC education, although we are very aware that this is controversial in many European countries.

It was challenging to compare our results with those reported in the literature. Many articles did not clarify sufficiently which *type* of teacher judgment accuracy was estimated (relative or absolute accuracy), on which *level of aggregation* accuracy was estimated (within groups of children or across groups, single-level, or multi-level models), or *how many parameters* were included in an estimation (multiple/simple regression or partial/simple correlations respectively). It would be helpful for the state of research if the methods used were explained in more detail so that it is actually possible to make meaningful comparisons. Documenting more methodological details—for example, in an electronic supplement—would also meet increasing open science requests, in particular, the possibility to reproduce results.

We noted a general lack of multi-level modeling in research on judgment accuracy. This may mean that meta-analyses and systematic reviews suffer from methodological limitations because the nested structure of judgments could not be taken into account (see also the corresponding remark in Südkamp et al., 2012). Moreover, the lack of multi-level modeling means that the advantage of simultaneously estimating absolute and relative teacher judgment accuracies in terms of a level (random intercepts) and a rank component (random slopes) only rarely has been utilized. Besides our study, we were able to identify only one other study (Bonefeld et al., 2020). We applied a multi-level framework that allowed us to assess judgment accuracy of EC teachers with a criterion on the within- and the between-group level while being able to include covariates. The Bayesian analysis had the additional benefit that individual effects on the teacher level were estimable and effect sizes in form of variance explained on various levels were readily available.

Another challenge in examining judgment accuracy was a lack of agreement about which level of correspondence between evaluations of teachers and skills of children can be regarded as “accurate.” To use our study as an example: 65.0% of the variance in KiDiT ratings of teachers were explained by MBK-0 scores of children (see Model 1). Does this amount reflect accurate judgments? We used statistical criteria (CIs) and a comparison of our correlation coefficients with other studies to evaluate the effect sizes. It would be helpful though to have contextualized benchmarks that would allow characterizing judgment accuracy similar to the effect sizes Cohen (1969) suggested. In addition, it has to be noted that the level component, as operationalized in the present study, only reflects how well a teacher would assess the skill of a child with an average skill level and does not address the full range of skills. There are approaches available when the Bayesian approach is used that could be further developed for this purpose. However, the viability and rationality of such approaches would need complex methodological considerations.

Besides such methodological considerations, the role of theory in EC research has to be stressed (Pianta et al., 2020). In our context, this applies in particular to conceptual work regarding predictors of the accuracy of EC teachers. Südkamp et al. (2012) developed a model of variables potentially related to judgment accuracy, which includes among others teacher characteristics. Our study indicates, however, no direct relation of teacher knowledge to accuracy. We interpret this result as a need to specify the teacher characteristics hypothesized to be relevant for judgment accuracy in more detail, for example with respect to their knowledge.

Another line of research useful with respect to judgment accuracy in play-based EC environments would be to examine whether systematic bias in the accuracy of teachers exists. This was not the topic of the present study. Previous studies with older children revealed that discrepancies between ratings of teachers and test scores of children may not be randomly distributed but related to the socio-economic background of children (Ready and Wright, 2011). It is an important follow-up RQ whether such bias also exists with respect to younger children. A range of context variables should be examined besides the socio-economic background of children, namely their gender, language background, and behavioral characteristics but also working conditions or neighborhood characteristics of EC teachers.

Finally, cross-sectional studies are dominating the research on judgment accuracy which means that we cannot always rule out reversed relations or third-variable explanations. It would therefore be important to carry out more longitudinal studies. Further progress can only be made with *a priori* planned study designs grounded in theory regarding the development of skills of children in the context of EC institutions.

## Data Availability Statement

The original contributions presented in the study are included in the article/Supplementary Material, further inquiries can be directed to the corresponding author.

## Ethics Statement

The studies involving human participants were reviewed and approved by the Federal Ministry of Education and Research in Germany (FKZ: 01PK15003A-C) and was part of the funding initiative KoKoHs (Modeling and Measuring Competencies in Higher Education—Validation and Methodological Innovations). Written informed consent to participate in this study was provided by the participants' legal guardian/next of kin.

## Author Contributions

GH conceived the idea of examining the preschool teacher's judgment accuracy, analyzed the data, wrote parts of the manuscript, and commented on the manuscript. SB wrote parts of the manuscript and commented on the manuscript. KE, LJ, and ME commented on the manuscript. All authors contributed to the article and approved the submitted version.

## Funding

This work was funded by the Federal Ministry of Education and Research, Germany, Grant Number 01PK15003A-C. The authors acknowledge support by the Open Access Publication Initiative of Freie Universität Berlin.

## Conflict of Interest

The authors declare that the research was conducted in the absence of any commercial or financial relationships that could be construed as a potential conflict of interest.

## Publisher's Note

All claims expressed in this article are solely those of the authors and do not necessarily represent those of their affiliated organizations, or those of the publisher, the editors and the reviewers. Any product that may be evaluated in this article, or claim that may be made by its manufacturer, is not guaranteed or endorsed by the publisher.
