# Supplementary material for: Absolute and Relative Judgment Accuracy: Early Childhood Teachers' Competence to Evaluate Children's Mathematical Skills
Source: Front Psychol. 2021 Oct 18;12:701730. doi: 10.3389/fpsyg.2021.701730 (PMC8558252; doi:10.3389/fpsyg.2021.701730)
Supplement: Supplementary file 3 [file Data_Sheet_3.PDF]

SI 1: MBK-0 scores and children's age in the analysis sample

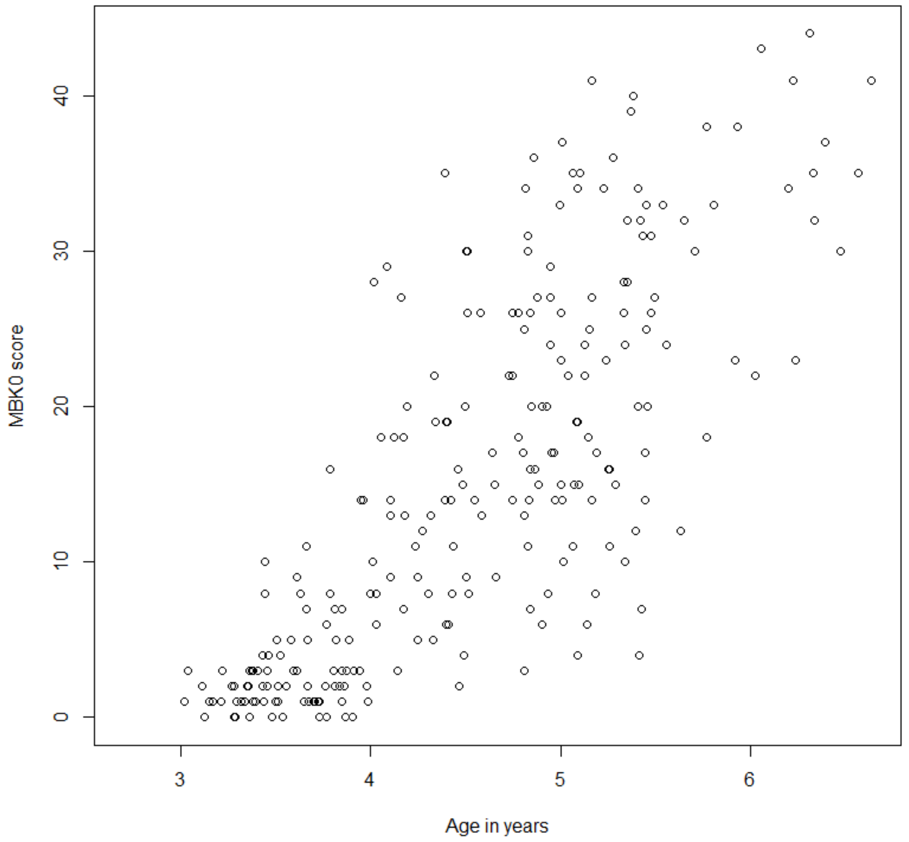

Figure SI1: Bivariate distribution of the MBK-0 scores and children's age in the analysis sample (N=252, as for 16 children the MBK0 score was missing).

SI 2: Example Items Teacher Tests

Example Item GPK (General Paedagogical Knowledge)

Two children (both 5 years old) are standing in the garden in front of a patch in which sunflowers were seeded to observe the process of growth of the plants. With which pedagogical concept would you explain the following dialogue?

Child 1: Why are the flowers here so little? We seeded some in plant pots on the windowsill back home and those are already bigger.

Child 2: They certainly grow longer.

Child 1: No, I and my father seeded them at the same day.

Child 2: However, something must be different on your windowsill than here in the garden, isnt'it?

Child 1: It is much warmer there than here outside because we turned the heater on.

Child2 : I think your flowers grow faster because it is warmer there than here in the garden.

Please indicate your answer with a cross.

- According to instructional learning
- According to interactional theory
- According to co-construction
- According to the theory of split attention

Example Item MCK (Mathematical Content Knowledge)

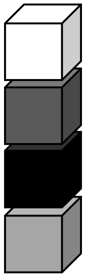

Chris has a blue, a green, a red and a yellow cube.
Chris wants to pile up a tower with the four cubes

Which arithmetic expression provides the number of possibilities of the different towers?
Please indicate your answer with a cross

- ○ $4 + 4 + 4 + 4$
- ○ $4 \cdot 4 \cdot 4 \cdot 4$
- ○ $4 \cdot 3 \cdot 2$
- ○ $4 + 3 + 2$

Example Item MPCK (Mathematical Pedagogical Content Knowledge)

You are playing a dice game with three children.

Please explain, in short, why their mathematical learning in the following field is fostered: **Numbers and operations (e.g., calculating)**

SI 3: Results of the stability analysis

Here, the results of the stability analysis are reported. We excluded the items for assessing geometry from the KiDiT, as the MBK0 does not assess this domain. The results from the Bayesian analysis are presented in the table. The results based on multiple imputation can be found in the Online Supplementary Material at [blinded for review].

Table SI3a

Model 1. Children's KiDiT scores multilevel-regressed on children's MBK-0 scores

Coefficient	Label	Estimate	*95%-CI*	*Standardized estimate*	*95%-CI*
Level-1 Residualvariance					
$\sigma^2_{\varepsilon_{ij}}$		0.224*	[0.184; 0.276]	--	--
Random Effects					
$\sigma^2_{u_{0j}}$	Intercept variance	0.256*	[0.142; 0.466]	1.000	--
$\sigma^2_{u_{1j}}$	Slope variance	0.067*	[0.020; 0.172]	1.000	--
$cov(u_{0j}, u_{1j})$;		-0.061	[-0.153; 0.012]	-.512	[-.874; .078]
Fixed Effects					
γ_{00}	Intercept	0.043	[-0.140; 0.233]	--	--
γ_{01}	Slope (MBK-0)	0.761*	[0.635; 0.885]	--	--
Within-level standardized effects averaged across teachers					
	KiDiT on MBK-0	--	--	.817*	[.359; .930]
	Residual variance	--	--	.332*	[.134; .866]

Note: * The 95%-credibility interval (CI) does not include the value of 0. Deviance Information Criterion (DIC) = 1119.82. Average R^2_{within}=.682 (95%-CI: [.593; .752]). $n_{teacher}$=39; $n_{children}$=268. Standardized estimates are given where appropriate. The within-level standardized effect of the regression of the KiDiT scores on the MBK-0 scores within one teacher's group and the residual variance are averaged over clusters (teachers).

Table SI3b

Model 2. Predicting the random intercepts (absolute judgment accuracy, level component) and random slopes (relative judgment accuracy, rank component) by teachers' test scores

Coefficient	Label	Estimate	95%-CI	Standardized Estimate	95%-CI
Level-1 Residual Variance					
$\sigma^2_{\varepsilon_{ij}}$		0.223*	[0.184 0.274]		
Random-Effects Variances and Covariances					
$\sigma^2_{u_{0j}}$		0.267*	[0.142; 0.521]		
$\sigma^2_{u_{1j}}$		0.078*	[0.021; 0.209]		
$cov(u_{0j}, u_{1j})$		-0.062	[-0.165; 0.028]	-0.479	[-0.885; 0.175]
Fixed Effects					
γ_{00}	Intercept	0.044	[-0.153; 0.238]	0.075	[-0.252; 0.410]
γ_{01}	MCK	0.025	[-0.029; 0.079]	0.361	[-0.395; 1.115]
γ_{02}	MPCK	-0.018	[-0.084; 0.044]	-0.211	[-0.969; 0.493]
γ_{03}	GPK	-0.029	[-0.089; 0.028]	-0.316	[-0.878; 0.317]
γ_{10}	Slope (MBK-0)	0.778*	[0.639; 0.923]	2.394*	[1.461; 4.212]
γ_{11}	MCK	-0.006	[-0.042; 0.032]	-0.164	[-1.169; 0.706]
γ_{12}	MPCK	0.020	[-0.025; 0.062]	0.417	[-0.507; 1.260]
γ_{13}	GPK	-0.015	[-0.058; 0.029]	-0.316	[-1.015; 0.544]
Covariance / Correlation of teachers' test scores on Level-2					
$cov(MCK, MPCK)$	MCK with MPCK	43.141*	[19.384; 94.849]	0.808*	[0.529; 0.924]

Coefficient	Label	Estimate	95%-CI	Standardized Estimate	95%-CI
$cov(MCK, GPK)$,	MCK with GPK	36.191*	[13.224; 80.161]	0.731*	[0.351; 0.890]
$cov(MPCK, GPK)$	MPCK with GPK	29.539*	[11.536; 63.421]	0.737*	[0.390; 0.887]
Within-level standardized effects across teachers					
	MBK-0 on KiDiT	--	--	0.810	[0.735; 0.862]
	Residual variances	--	--	0.323	[0.248; 0.410]

Note: * The 95%-credibility interval (CI) does not contain the value of 0. Deviance Information Criterion (DIC) = 1591.09; Average R^2_{within}=.677 (95%-CI: [.590, .751]); $R^2_{\beta_{0j(between)}}$=.173 (95%-CI: [.019, .474]); $R^2_{\beta_{1j(between)}}$=.231 (95%-CI: [.026, .636]); $n_{teacher}$=39; $n_{children}$=268. Standardized estimates are given where appropriate. The standardized within-level effects and residual variances are averaged over clusters (teachers).
